# Development of artificial neural networks for early prediction of intestinal perforation in preterm infants

**DOI:** 10.1038/s41598-022-16273-5

**Published:** 2022-07-15

**Authors:** Joonhyuk Son, Daehyun Kim, Jae Yoon Na, Donggoo Jung, Ja-Hye Ahn, Tae Hyun Kim, Hyun-Kyung Park

**Affiliations:** 1 Department of Pediatric Surgery, Hanyang University College of Medicine, 222 Wangsimni-ro, Seongdong-gu, Seoul, 04763 Korea; 2 Department of Artificial Intelligence, Hanyang University, 222 Wangsimni-ro, Seongdong-gu, Seoul, 04763 Korea; 3 Department of Pediatrics, Hanyang University College of Medicine, 222 Wangsimni-ro, Seongdong-gu, Seoul, 04763 Korea; 4 Department of Computer Science, Hanyang University, 222 Wangsimni-ro, Seongdong-gu, Seoul, 04763 Korea

**Keywords:** Infant necrotizing enterocolitis, Machine learning

## Abstract

Intestinal perforation (IP) in preterm infants is a life-threatening condition that may result in serious complications and increased mortality. Early Prediction of IP in infants is important, but challenging due to its multifactorial and complex nature of the disease. Thus, there are no reliable tools to predict IP in infants. In this study, we developed new machine learning (ML) models for predicting IP in very low birth weight (VLBW) infants and compared their performance to that of classic ML methods. We developed artificial neural networks (ANNs) using VLBW infant data from a nationwide cohort and prospective web-based registry. The new ANN models, which outperformed all other classic ML methods, showed an area under the receiver operating characteristic curve (AUROC) of 0.8832 for predicting IP associated with necrotizing enterocolitis (NEC-IP) and 0.8797 for spontaneous IP (SIP). We tested these algorithms using patient data from our institution, which were not included in the training dataset, and obtained an AUROC of 1.0000 for NEC-IP and 0.9364 for SIP. NEC-IP and SIP in VLBW infants can be predicted at an excellent performance level with these newly developed ML models. https://github.com/kdhRick2222/Early-Prediction-of-Intestinal-Perforation-in-Preterm-Infants.

## Introduction

Intestinal perforation (IP) in preterm infants is a life-threatening condition that requires urgent surgical intervention. Delayed diagnosis of IP can lead to serious complications and even death in certain patients. There are two major causes of IP in premature infants: necrotizing enterocolitis (NEC) and spontaneous intestinal perforation (SIP)^1–5^. NEC and SIP are acquired diseases, which means that IP occurs due to the actions of various risk factors. IP associated with NEC (NEC-IP) is an advanced stage of NEC in which NEC has progressed to a severe state. On the other hand, SIP can occur unexpectedly without preceding symptoms or signs^6^. Many previous studies have investigated the risk factors and pathogenesis of these two types of IP. According to the literature, possible risk factors include prematurity, low birth weight, perinatal use of medications (ibuprofen, steroids, indomethacin, and inotropic agents), maternal chorioamnionitis, patent ductus arteriosus (PDA), and high-grade intraventricular hemorrhage (IVH grade III or IV)^5,7–22^. Despite these efforts, due to the nature of retrospective studies with a small number of patients, there is thus far no consensus concerning risk factors. Furthermore, the pathogenesis of IP in premature infants is still not clearly understood; as a result, neonatologists and pediatric surgeons have difficulty predicting and preventing the occurrence of NEC-IP and SIP^23–26^.

Recently, artificial intelligence (AI) technologies have been used to solve various medical problems, including diagnosing diseases, choosing treatments, and predicting the risks and progression of diseases^27–30^. Machine learning (ML) algorithms, specifically artificial neural networks (ANNs), allow more accurate estimation of complex patterns through learning from training data and are able to predict which patients will develop a disease as an early and supportive diagnostic tool for clinicians. To date, only a few studies have used ML algorithms to predict IP in premature infants. Irles et al.^31^ reported an ML model predicting NEC-IP, and Lure et al.^32^ reported an ML model to differentiate NEC-IP and SIP prior to surgical interventions. Lin^33^ et al. reported an ML technique for individualized NEC risk scores using intestinal microbiota data. However, despite these efforts, studies concerning ML technologies in neonatal IP are still lacking, and there are no definitive tools to predict NEC-IP and SIP in advance. Therefore, our study aimed to develop our own ML models for predicting IP in very low birth weight (VLBW) infants using a nationwide cohort dataset and to compare the performance of the new models with that of classic ML models. Furthermore, to ensure that our novel predictive ML models were valid in real neonatal intensive care (NICU) settings, we tested new ML models with individual cases from our institution and assessed whether these new models achieved high predictive accuracy.

## Results

### Baseline characteristics of the patients

The baseline characteristics of the infants are shown in Table 1. Of the 12,555 patients, 521 patients had NEC-IP (4.1%), and 208 patients had SIP (1.7%). Three groups of VLBW infants were compared for analysis: (A) control group without NEC-IP or SIP, (B) patients with NEC-IP, and (C) patients with SIP. Factors such as low gestational age, low birth weight, need for resuscitation at delivery, respiratory distress syndrome (RDS) and administration of surfactant, use of certain medications (steroids, ibuprofen, and inotropes), hypotension, IVH grade III/IV, sepsis, and PDA on treatment were significantly associated with IP, including both NEC-IP and SIP.

**Table 1:** Clinical characteristics of the patients.

	A	B	C	p-value	
	No perforation (n = 11,826)	NEC-IP (n = 521)	SIP (n = 208)	A vs. B	A vs. C
Gestational age (weeks, mean ± SD)	28.58 ± 3.02	25.84 ± 2.41	26.25 ± 2.23	< 0.001	< 0.001
Birth weight (g, mean ± SD)	1092.38 ± 284.05	820.03 ± 249.41	858.38 ± 248.15	< 0.001	< 0.001
Sex-male—n (%)	5919 (50.1)	285 (54.7)	130 (62.5)	0.038	< 0.001
Maternal chorioamnionitis—n (%)	3426 (29.0)	162 (31.1)	64 (30.8)	0.296	0.571
PROM—n (%)	4073 (34.4)	196 (37.6)	80 (38.5)	0.135	0.227
Antenatal steroid use—n (%)	9341 (79.0)	414 (79.5)	169 (81.3)	0.794	0.427
Resuscitation at delivery—n (%)	10,472 (88.6)	489 (93.9)	204 (98.1)	< 0.001	< 0.001
RDS—n (%)	9080 (76.8)	487 (93.5)	198 (95.2)	< 0.001	< 0.001
Surfactant use—n (%)	9114 (77.1)	489 (93.9)	200 (96.2)	< 0.001	< 0.001
Steroid use—n (%)	2589 (21.9)	198 (38.0)	91 (43.8)	< 0.001	< 0.001
Indomethacin use—n (%)	54 (0.5)	8 (1.5)	2 (1.0)	0.001	0.252
Ibuprofen use—n (%)	3539 (29.9)	232 (44.5)	83 (39.9)	< 0.001	0.002
Hypotension—n (%)	2838 (24.0)	325 (62.4)	130 (62.5)	< 0.001	< 0.001
Inotropic use—n (%)	548 (4.6)	59 (11.3)	26 (12.5)	< 0.001	< 0.001
IVH grade 3,4—n (%)	934 (7.9)	146 (28.0)	48 (23.1)	< 0.001	< 0.001
Sepsis—n (%)	2224 (18.8)	266 (51.1)	91 (43.8)	< 0.001	< 0.001
PDA on medication—n (%)	3615 (30.6)	241 (46.3)	87 (41.8)	< 0.001	< 0.001
PDA ligation—n (%)	1145 (9.7)	142 (27.3)	48 (23.1)	< 0.001	< 0.001

*NEC-IP* intestinal perforation associated with necrotizing enterocolitis, *SIP* spontaneous intestinal perforation, *SD* standard deviation, *PROM* premature rupture of membranes, *RDS* respiratory distress syndrome, *IVH* intraventricular hemorrhage, *PDA* patent ductus arteriosus.

### Proposed machine learning algorithms

In the work of Irles et al.^31^, ML-based approaches were proposed to predict NEC values, and our neural approaches produced promising NEC prediction results using a dataset that included 852 positive cases. However, it is more difficult to collect data for NEC-IP and SIP than for NEC; thus, the number of datasets, especially for positive cases, is very limited (521 and 208 positive cases for NEC-IP and SIP, respectively). To solve this problem, we utilized relevant information from a neural network trained for NEC prediction to elevate the diagnostic accuracy of SIP and NEC-IP.

**Figure 1:**
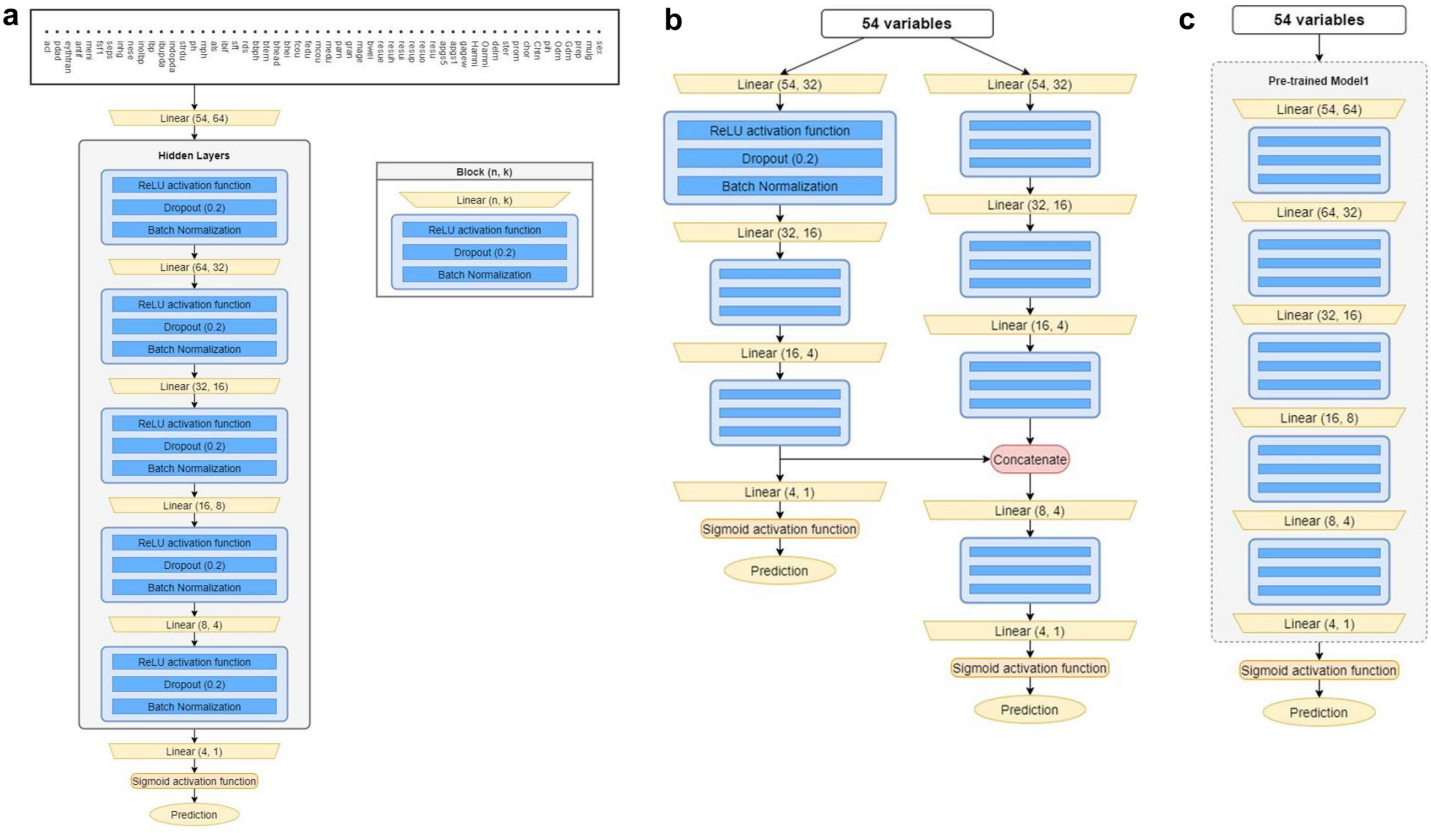
Architecture of machine learning models. (**a**) Model 1 is a baseline neural network based on the conventional multilayer perceptron (MLP) architecture. (**b**) Model 2 is composed of two different MLPs. One branch predicts NEC, and the other branch predicts either NEC-IP or SIP. The feature vectors from the 3rd layer of the network for NEC are concatenated with the 4th layer of the MLP branch for NEC-IP and SIP. (**c**) Model 3 has the same network architecture as that of Model 1. Pretrained Model 1 for NEC was further fine-tuned to estimate NEC-IP/SIP.

In this study, we introduced several deep neural networks for predicting NEC, NEC-IP, and SIP in infants by taking a 54-dimensional input vector as the network input, and the output of each network was a single value for binary classification problems. Specifically, we first developed our baseline neural network (Model 1) based on the conventional multilayer perceptron (MLP) architecture and then trained Model 1 to predict NEC, NEC-IP, and SIP separately. For ANNs, it is well known that simply adding layers can lead to performance improvement by enforcing more nonlinearity; however, it causes the overfitting problem when the training dataset is insufficient. Therefore, we stacked the layers more deeply than the conventional models in diagnosis, as introduced in a previously published study^31^, but determined the hyperparameters (e.g., the number of channels) carefully and added more advanced techniques, such as batch normalization^34^ and drop-out^35^, to avoid the overfitting problem and facilitate stable training. Specifically, Model 1 is a binary classifier that is composed of 5 hidden layers; each layer is formed from a block (Fig. 1a) arranged with an activation function, batch normalization and dropout. It takes a 54-dimensional vector as an input and, after the application of all layers, renders feature vectors of dimension. Model 1 is a typical neural approach and can produce promising results when a large number of datasets (e.g., NEC) are available. However, the outcomes from Model 1 can be vulnerable in real-world scenarios where the training dataset is insufficient.

Therefore, we attempted to further improve our baseline Model 1 to predict NEC-IP and SIP more accurately since the lack of data problem for NEC-IP and SIP is more serious than that for NEC. To alleviate this problem, we developed a new approach that transferred information from the network to predict NEC values to help estimate NEC-IP and SIP. Note that transfer learning in the field of deep learning is one of the most widely used approaches to solve lack of data problems^36–40^. Therefore, we present additional models (Model 2 and Model 3) that can exploit information achieved from the NEC dataset to predict SIP and NEC-IP.

Specifically, Model 2 is composed of two different MLPs. One branch predicts NEC, and the other branch predicts either SIP or NEC-IP (Fig. 1b). Notably, at the 4th layer of the MLP branch for NEC-IP and SIP in Model 2, feature vectors from the third layer of the network for NEC are fed as an additional input. By concatenating the feature vectors from NEC, we can utilize the information for NEC in predicting NEC-IP/SIP.

Unlike that of Model 2, the network architecture of Model 3 is the same as that of Model 1. We employed conventional transfer learning to utilize information from NEC to estimate NEC-IP/SIP and fine-tuned the pretrained Model 1 to the specific NEC-IP/SIP datasets (Fig. 1c).

### Comparison of performance between classic ML models and proposed ANN models

We provide prediction results in Table 2 to compare traditional ML models with our neural approaches. We observed that the proposed neural approach (Model 1) outperformed traditional learning-based methods in terms of area under the receiver operating characteristic curve (AUROC) scores for all cases (NEC, NEC-IP, and SIP).

**Table 2:** Model performance of classic ML models for predicting NEC, NEC-IP, and SIP.

AUROC	NEC	NEC-IP	SIP
Linear SVM	0.7632	0.8618	0.8162
Radial SVM	0.7567	0.8195	0.7481
Logistic regression	0.7641	0.8644	0.8044
K-NN	0.6229	0.6337	0.5759
Decision tree	0.5145	0.5377	0.5004
XGBoost	0.6758	0.7748	0.7452
LightGBM	0.7087	0.7758	0.7477
Random forest	0.7495	0.8051	0.7687
MLP (model 1)	0.8128	0.8665	0.8498

*ML* machine learning, *NEC* necrotizing enterocolitis, *NEC-IP* intestinal perforation associated with necrotizing enterocolitis, *SIP* spontaneous intestinal perforation, *SVM* support vector machine, *K-NN* k-nearest neighbor, *XGBoost* extreme gradient boosting, *GBM* gradient boosting machine learning*, LightGBM* light gradient boosting machine learning, *MLP* multilayer perceptron.

Moreover, our extended networks (i.e., Model 2 and Model 3), which we designed to mitigate the lack of data problem for NEC-IP (521 positive cases) and SIP (208 positive cases), showed improved results in predicting NEC-IP and SIP over the baseline network (Model 1).

**Figure 2:**
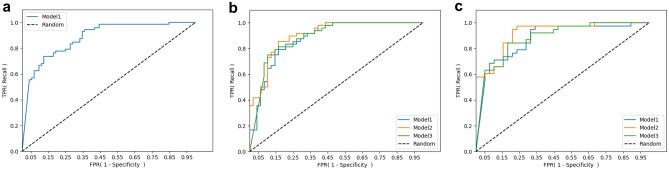
Receiver operating characteristic curves of proposed ML models for (**a**) NEC prediction, (**b**) NEC-IP prediction, and (**c**) SIP prediction.

In particular, compared to Model 1, which was trained on the NEC dataset, our proposed methods for NEC-IP and SIP (Model 2 and Model 3) exhibited improvements. Notably, Model 2 directly utilizes features distilled from Model 1, Model 3 fine-tunes Model 1 for the prediction of either NEC-IP or SIP, and Model 2 outperforms Model 3 in terms of AUROC, as shown in Table 3 and Fig. 2. The performance metrics of these models using balanced validation dataset are described in Supplementary Tables 1 and 2. This performance improvement achieved in Model 2 and Model 3 indicates that information extracted to predict NEC can also be used to predict NEC-IP and SIP more accurately. Moreover, the proposed direct feature distillation of Model 2 rather than the conventional fine-tuning approach (i.e., Model 3) can be a recommendable option for addressing problems with limited data.

**Table 3:** Performance of the proposed ANN models in predicting NEC, NEC-IP, and SIP.

	Model 1	Model 2	Model 3
**AUROC**			
NEC	0.8128	–	–
NEC-IP	0.8665	0.8832	0.8692
SIP	0.8498	0.8797	0.8633
**F1-score**^**a**^			
NEC	0.7701	–	–
NEC-IP	0.7181	0.8093	0.7273
SIP	0.8059	0.8204	0.8041

*ANN* artificial neural network, *NEC* necrotizing enterocolitis, *NEC-IP* intestinal perforation associated with necrotizing enterocolitis, *SIP* spontaneous intestinal perforation, *AUROC* area under the receiver operating characteristic curve.

^a^Scores were found with balanced validation dataset. Positive cases were oversampled.

### Application of the new ANN models in a real clinical environment

**Figure 3:**
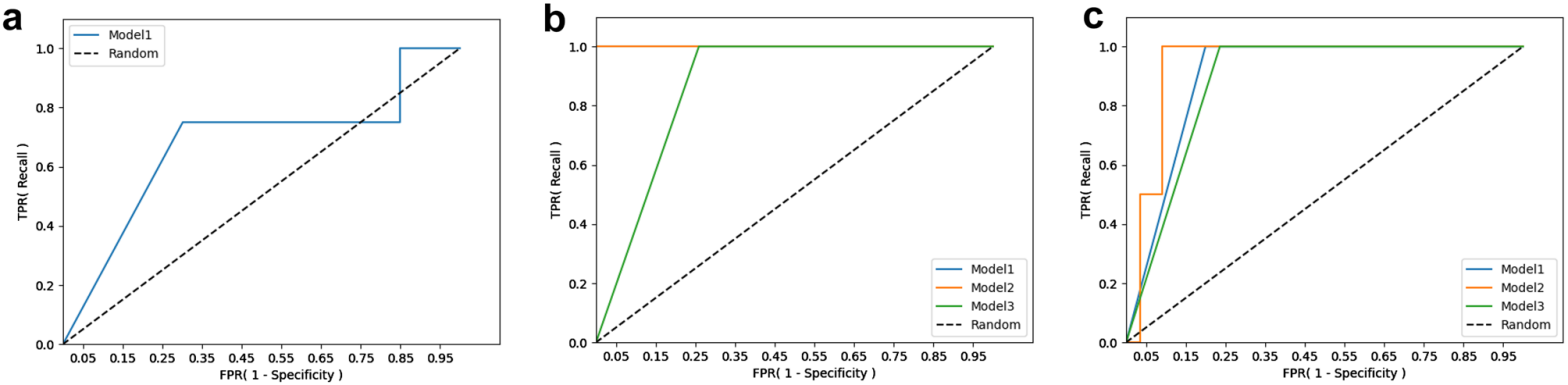
Receiver operating characteristic curves of proposed ML models from 57 test cases. (**a**) NEC prediction, (**b**) NEC-IP prediction, and (**c**) SIP prediction.

To assess the feasibility of the algorithm in a real clinical environment, we tested our newly developed algorithms using the patient data from our institution, which were not included in the training dataset. A total of 57 VLBW infants who were born at our hospital between 2019 and 2020 were included in this test analysis. Among the three ANN models, Model 2 achieved the highest AUROC scores: 1.0000 for the prediction of NEC-IP and 0.9364 for the prediction of SIP (Table 4 and Fig. 3).

**Table 4:** Test results for 57 cases within a real NICU environment.

	Model 1	Model 2	Model 3
**AUROC**			
NEC	0.6745	–	–
NEC-IP	1.0000	1.0000	0.8704
SIP	0.9000	0.9364	0.8818
**F1-score**^**a**^			
NEC	0.6903	–	–
NEC-IP	0.8571	0.9076	0.8710
SIP	0.7241	0.7179	0.7925

*NICU* neonatal intensive care unit, *AUROC* area under the receiver operating characteristic curve, *NEC* necrotizing enterocolitis, *NEC-IP* intestinal perforation associated with necrotizing enterocolitis, *SIP* spontaneous intestinal perforation.

^a^Scores were found with balanced validation dataset. Positive cases were oversampled.

## Discussion

Our novel ML algorithms predicted NEC-IP and SIP in VLBW infants with favorable AUROC scores, outperforming all other classic ML algorithms. One of our algorithms exhibited an AUROC score of 1.0000 for predicting NEC-IP and 0.9364 for predicting SIP in real clinical settings. Our study shows the integration of a vast nationwide dataset with ML, and the resulting model can be used to predict the possibility of specific medical conditions in patients who may not perfectly represent the signs and symptoms of the disease.

Among preterm infants in the NICU, various medical problems exist at the same time, and multidisciplinary collaboration is required to make medical decisions for these patients. IP is one of the most devastating medical conditions that occurs in the NICU. Early diagnosis, swift judgment, and prompt surgical intervention are required to prevent severe complications and poor outcomes^41^. However, as we explained earlier, predicting the occurrence of IP is difficult. By running this algorithm, it is possible to analyze every preterm infant who is admitted to the NICU and predict each patient’s likelihood of developing NEC-IP and SIP. Thus, early predictions of these serious medical conditions could provide clinicians with a much more stable management environment, enabling them to make better treatment decisions.

In recent years, AI and big data have been increasingly integrated into medicine because it is difficult for the unaided human clinician to acquire all the latest published knowledge, as is required by modern evidence-based medicine^30,42^. AI is an important resource for medical research in that it can efficiently process large amounts of data. Additionally, AI can produce consistent and unbiased results without fatigue. Several studies in healthcare research have reported sufficient or even better risk prediction by AI methods compared to existing models^43–47^. Especially these days, in solving difficult problems with big data which have complex distribution, neural approaches exhibit excellent performance. Since neural networks can have non-linearity from adding layers and fine-tune parameters by transfer learning^36,40^, they can get the upper hand in complex tasks. To sum up, ANN is an appropriate model for IP prediction, as it can handle imbalanced big data efficiently. However, even if the overall cohort is large, diseases with low prevalence always suffer from a lack of data. Due to the character of ML, it can produce excellent results only with a large amount of training data. Thus, applying ML to diseases with low prevalence remains a challenge^48,49^. To overcome the data imbalance problem, several studies have applied data processing techniques such as oversampling^50^ and undersampling^51^ as we did in our ANN models.

To further improve the performance of our models, we modified them by adding another branch or pretraining it based on an algorithm that predicts other relevant disease with higher prevalence in order to help the model predict the target disease more accurately. As a result of these adjustments, the modified algorithms (Model 2 and 3) achieved better performance than the original model. According to previous studies, NEC-IP and SIP are regarded as separate disease entities, and the pathogenesis of SIP does not appear to correlate with that of NEC^5,6,52–55^. Notably, however, training of NEC prediction improved not only the accuracy of NEC-IP prediction but also the accuracy of SIP prediction. These results show that pre-ML training with more prevalent medical conditions can help AI predict the occurrence of the target disease more accurately. Our study also highlights that it is necessary to customize the algorithm for each disease to apply an ML model in real clinical settings, especially if the disease is rare.

Although our study showed a favorable outcome, it had its share of limitations. First, a limited number of factors are included because only data collected from the Korean Neonatal Network (KNN) were used. It is expected that the collection of further IP-related data, such as clinical symptoms, vital signs, and radiologic findings, will enable the model to produce better results. In addition, the limitations of AI studies, such as representation, homogeneity, and accuracy, were observed in this study. Another limitation is that it is difficult to determine how AI methods generate results due to the nature of self-extracted data from large datasets^49,56,57^.

In conclusion, we developed our own ANN models to predict IP early in VLBW infants, and these new models achieved higher accuracy than classic ML algorithms. To our knowledge, this is the first study to develop an ML model to predict both NEC-IP and SIP using nationwide VLBW infant data. In addition, the newly proposed ANN models showed excellent performance within real NICU clinical settings. When more clinical data, such as vital signs, radiologic findings, biomarkers, and laboratory results, are gathered, we believe that a more accurate ML model will be developed, thereby achieving early prediction of these serious medical conditions and better clinical outcomes for VLBW infants.

## Methods

### Data collection

We derived data from infants registered in the KNN, a nationwide prospective cohort registry of VLBW infants^58^. Their clinical data were collected from 74 participating NICUs across the country and analyzed retrospectively for this study. Prior to participation in the KNN registry, informed consent was obtained from the parents of each infant, and all methods were carried out following relevant guidelines. This study was approved by the Hanyang University Institutional Review Board (IRB No. 2013-06-025-043).

The cohort comprised 12,555 VLBW infants born between January 5, 2013, and December 31, 2018, weighing less than 1500×*g*.

### Disease definitions

NEC was defined according to Bell's modified staging grade ≥ II. NEC-IP was diagnosed when patients with NEC underwent any kind of abdominal surgical intervention (peritoneal drainage or laparotomy). SIP was defined when the patients underwent surgical intervention due to IP and the surgeon found no predisposing causes, such as NEC, intestinal atresia, or meconium plug. The full list of 54 variables used in ML analysis is shown in Supplementary Table 3.

### Comparisons of baseline characteristics

A total of 54 variables, including various maternal and perinatal factors, were collected for ML. Among them, 18 clinical factors that were proposed as possible risk factors for either NEC-IP or SIP in previous studies were analyzed using conventional statistical methods. Student’s t-test was performed to analyze the continuous variables, and the chi-squared test was used to analyze categorical variables. Statistical significance was set at *P* < 0.05. The Statistical Package for the Social Sciences version 22.0 for Windows software program (IBM Corp., Armonk, NY, USA) was used in all statistical analyses.

### Data preprocessing

**Figure 4:**
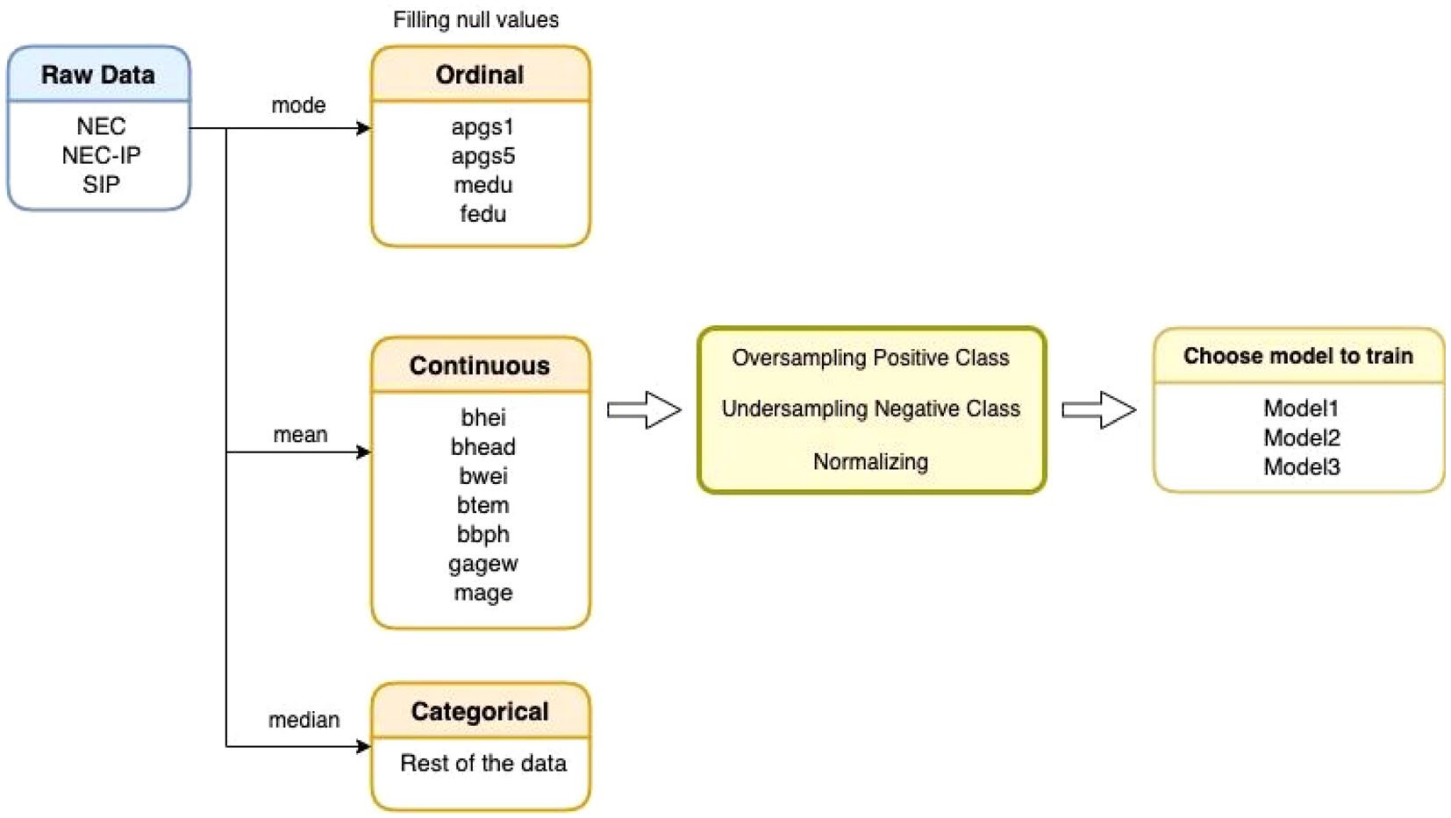
Flowchart of data processing. Input values were categorized into ordinal, continuous and categorical types. To solve the data imbalance problem, oversampling and undersampling technique were applied. Then, the data were normalized.

Our dataset was composed of 12,555 infants in total; we divided them into training and evaluation datasets (Table 5). Moreover, to facilitate the network training procedure, a data preprocessing step was applied (Fig. 4). First, to solve the missing data problem in the given dataset, we imputed the missing (null) values with plausible values. Specifically, input values were categorized into ordinal, continuous and categorical types. In the case of ordinal inputs, we imputed the null values with the mode (most frequently occurring) values, and in the case of continuous inputs, we imputed the null values with the mean values. Finally, missing values of categorical inputs were replaced with the median values. After recovering the data, we mapped the dataset between 0 and 1 by using min–max normalization. Finally, to mitigate the data imbalance problem (e.g., 11,703 negative and 852 positive cases for NEC in Table 5), which is a common intrinsic feature of disease datasets, we oversampled the smaller category (positive cases) and undersampled the bigger category (negative cases), as suggested in previous studies^59,60^.

**Table 5:** Size of training and evaluation datasets.

	Training dataset		Evaluation dataset		Total
	Negative	Positive	Negative	Positive	
NEC	10,000	770	1703	82	12,555
NEC-IP		430	2035	91	
SIP		170	2348	38	

*NEC* necrotizing enterocolitis, *NEC-IP* intestinal perforation associated with necrotizing enterocolitis, *SIP* spontaneous intestinal perforation.

### Training

We used the binary cross entropy (BCE) loss to train Model 1, and Model 1 was separately trained to predict NEC, NEC-IP, and SIP. For Model 3, pretrained Model 1 for NEC was further fine-tuned, and the separately updated parameters using the BCE loss were used to predict NEC-IP and SIP.

Unlike Model 1 and Model 3, Model 2 was trained in a two-stage manner. In the first stage, the left branch of the network (Fig. 1b) was trained for NEC with the BCE loss. Then, the BCE loss as well as the BCE for NEC in the left branch were used to jointly optimize the network for either NEC-IP or SIP. In both training steps, oversampling and undersampling technique were used to alleviate the data imbalance problem. We employed the two-stage training scheme rather than using the one-shot joint training approach, as we could retain more stability during the training from the two-stage approach.

To train the three proposed models (i.e., Model 1, Model 2, Model 3), the Adam optimizer^61^ with a learning rate of 0.0001 was used for loss minimization. We used a dropout rate of 0.2^35^ and a batch size of 128^34^. Instead of fixing the number of iterations, we stopped the training using the early stopping technique to avoid the overfitting problem. When the loss increased seven times in a row, we stopped the training (i.e., early stop) and took the parameters just prior to the loss increase. The models were implemented in PyTorch with Python^62^, and the Scikit-learn library^63^ was used to evaluate the results (e.g., AUROC, F1-Score, and ROC curve).

## Supplementary Information

Supplementary Tables.

## Data Availability

According to the Korean Neonatal Network (KNN) Publication Ethics Policy, all information about patients is confidential. The information contained in the data must be protected as confidential, and only available to individuals who have access for the permitted research activity.

## References

[1] Hunter CJ, Chokshi N, Ford HR (2008). Evidence vs experience in the surgical management of necrotizing enterocolitis and focal intestinal perforation. J. Perinatol..

[2] Karila K, Anttila A, Iber T, Pakarinen M, Koivusalo A (2018). Outcomes of surgery for necrotizing enterocolitis and spontaneous intestinal perforation in Finland during 1986–2014. J. Pediatr. Surg..

[3] Vongbhavit K, Underwood MA (2017). Intestinal perforation in the premature infant. J. Neonatal Perinatal Med..

[4] Zozaya C, Shah J, Pierro A (2021). Neurodevelopmental and growth outcomes of extremely preterm infants with necrotizing enterocolitis or spontaneous intestinal perforation. J. Pediatr. Surg..

[5] Shah J, Singhal N, da Silva O (2015). Intestinal perforation in very preterm neonates: Risk factors and outcomes. J. Perinatol..

[6] Pumberger W, Mayr M, Kohlhauser C, Weninger M (2002). Spontaneous localized intestinal perforation in very-low-birth-weight infants: A distinct clinical entity different from necrotizing enterocolitis. J. Am. Coll. Surg..

[7] Ragouilliaux CJ, Keeney SE, Hawkins HK, Rowen JL (2007). Maternal factors in extremely low birth weight infants who develop spontaneous intestinal perforation. Pediatrics.

[8] Rayyan M, Myatchin I, Naulaers G, Ali Said Y, Allegaert K, Miserez M (2018). Risk factors for spontaneous localized intestinal perforation in the preterm infant. J. Matern. Fetal Neonatal Med..

[9] Rose AT, Patel RM (2018). A critical analysis of risk factors for necrotizing enterocolitis. Semin. Fetal Neonat. M..

[10] Samuels N, van de Graaf RA, de Jonge RCJ, Reiss IKM, Vermeulen MJ (2017). Risk factors for necrotizing enterocolitis in neonates: A systematic review of prognostic studies. BMC Pediatr..

[11] Youn YA, Kim EK, Kim SY (2015). Necrotizing enterocolitis among very-low-birth-weight infants in Korea. J. Korean Med. Sci..

[12] Arnautovic TI, Longo JL, Trail-Burns EJ, Tucker R, Keszler M, Laptook AR (2021). Antenatal risk factors associated with spontaneous intestinal perforation in preterm infants receiving postnatal indomethacin. J. Pediatr..

[13] Kandraju H, Kanungo J, Lee KS (2021). Association of co-exposure of antenatal steroid and prophylactic indomethacin with spontaneous intestinal perforation. J. Pediatr..

[14] Stavel M, Wong J, Cieslak Z, Sherlock R, Claveau M, Shah PS (2017). Effect of prophylactic indomethacin administration and early feeding on spontaneous intestinal perforation in extremely low-birth-weight infants. J. Perinatol..

[15] Yilmaz Y, Kutman HG, Ulu HO (2014). Preeclampsia is an independent risk factor for spontaneous intestinal perforation in very preterm infants. J. Matern. Fetal Neonatal Med..

[16] Houben CH, Feng XN, Chan KWE, Mou JWC, Tam YH, Lee KH (2017). Spontaneous intestinal perforation: The long-term outcome. Eur. J. Pediatr. Surg..

[17] Prasad U, Mohnani A, Hussain N (2021). Spontaneous intestinal perforation associated with premature twin infants. J. Neonatal Perinatal Med..

[18] Maheshwari A, Patel RM, Christensen RD (2018). Anemia, red blood cell transfusions, and necrotizing enterocolitis. Semin. Pediatr. Surg..

[19] Wang ZL, An Y, He Y (2020). Risk factors of necrotizing enterocolitis in neonates with sepsis: A retrospective case-control study. Int. J. Immunopathol. Pharmacol..

[20] Tatli MM, Kumral A, Duman N, Demir K, Gurcu O, Ozkan H (2004). Spontaneous intestinal perforation after oral ibuprofen treatment of patent ductus arteriosus in two very-low-birthweight infants. Acta Paediatr..

[21] Ndour D, Bouamari H, Berthiller J, Claris O, Plaisant F, Nguyen KA (2020). Adverse events related to ibuprofen treatment for patent ductus arteriosus in premature neonates. Arch. Pediatr..

[22] El Manouni El Hassani S, Niemarkt HJ, Derikx JPM (2021). Predictive factors for surgical treatment in preterm neonates with necrotizing enterocolitis: A multicenter case-control study. Eur. J. Pediatr..

[23] Eaton S, Rees CM, Hall NJ (2017). Current research on the epidemiology, pathogenesis, and management of necrotizing enterocolitis. Neonatology.

[24] Neu J (2014). Necrotizing enterocolitis: The mystery goes on. Neonatology.

[25] Frost BL, Modi BP, Jaksic T, Caplan MS (2017). New medical and surgical insights into neonatal necrotizing enterocolitis: A review. JAMA Pediatr..

[26] Cho SX, Rudloff I, Lao JC (2020). Characterization of the pathoimmunology of necrotizing enterocolitis reveals novel therapeutic opportunities. Nat. Commun..

[27] Krittanawong C, Bomback AS, Baber U, Bangalore S, Messerli FH, Wilson Tang WH (2018). Future direction for using artificial intelligence to predict and manage hypertension. Curr. Hypertens. Rep..

[28] Obermeyer Z, Emanuel EJ (2016). Predicting the future: Big data, machine learning, and clinical medicine. N. Engl. J. Med..

[29] Zhang Z, Liu J, Xi J, Gong Y, Zeng L, Ma P (2021). Derivation and validation of an ensemble model for the prediction of agitation in mechanically ventilated patients maintained under light sedation. Crit. Care Med..

[30] Na JY, Kim D, Kwon AM (2021). Artificial intelligence model comparison for risk factor analysis of patent ductus arteriosus in nationwide very low birth weight infants cohort. Sci. Rep..

[31] Irles C, Gonzalez-Perez G, Carrera Muinos S (2018). Estimation of neonatal intestinal perforation associated with necrotizing enterocolitis by machine learning reveals new key factors. Int. J. Environ. Res. Public Health..

[32] Lure AC, Du X, Black EW (2020). Using machine learning analysis to assist in differentiating between necrotizing enterocolitis and spontaneous intestinal perforation: A novel predictive analytic tool. J. Pediatr. Surg..

[33] Lin YC, Salleb-Aouissi A, Hooven TA (2022). Interpretable prediction of necrotizing enterocolitis from machine learning analysis of premature infant stool microbiota. BMC Bioinform..

[34] Ioffe, S. & Szegedy, C. Batch normalization: Accelerating deep network training by reducing internal covariate shift. in *Proceedings of the 32nd International Conference on Machine Learning,* vol 37, 448–456 (PMLR, 2015).

[35] Srivastava N, Hinton G, Krizhevsky A, Sutskever I, Salakhutdinov R (2014). Dropout: A simple way to prevent neural networks from overfitting. J. Mach. Learn. Res..

[36] Pan SJ, Yang QA (2010). A survey on transfer learning. IEEE Trans. Knowl. Data Eng..

[37] Rawat W, Wang ZH (2017). Deep convolutional neural networks for image classification: a comprehensive review. Neural Comput..

[38] Bengio, Y. Deep learning of representations for unsupervised and transfer learning. in *Proceedings of ICML workshop on unsupervised and transfer learning*. (JMLR Workshop and Conference Proceedings, 2012).

[39] Tan CQ (2018). A survey on deep transfer learning. Artif. Neural Netw. Mach. Learn..

[40] Zhuang FZ (2021). A comprehensive survey on transfer learning. Proc. IEEE.

[41] Bethell GS, Knight M, Hall NJ (2021). Surgical necrotizing enterocolitis: Association between surgical indication, timing, and outcomes. J. Pediatr. Surg..

[42] Barry-Jester AM, Casselman B, Goldstein D (2015). The new science of sentencing. Marshall Project..

[43] Alaa AM, Bolton T, Di Angelantonio E, Rudd JHF, van der Schaar M (2019). Cardiovascular disease risk prediction using automated machine learning: A prospective study of 423,604 UK Biobank participants. PLoS ONE.

[44] Jhee JH, Lee S, Park Y (2019). Prediction model development of late-onset preeclampsia using machine learning-based methods. PLoS ONE.

[45] Lee Y, Ryu J, Kang MW (2021). Machine learning-based prediction of acute kidney injury after nephrectomy in patients with renal cell carcinoma. Sci. Rep..

[46] Safavi KC, Khaniyev T, Copenhaver M (2019). Development and validation of a machine learning model to aid discharge processes for inpatient surgical care. JAMA Netw. Open..

[47] Weng SF, Reps J, Kai J, Garibaldi JM, Qureshi N (2017). Can machine-learning improve cardiovascular risk prediction using routine clinical data?. PLoS ONE.

[48] Murdoch WJ, Singh C, Kumbier K, Abbasi-Asl R, Yu BJ (2019). Interpretable Machine Learning: Definitions, Methods, and Applications.

[49] Rajkomar A, Dean J, Kohane I (2019). Machine learning in medicine. N. Engl. J. Med..

[50] Goh KH, Wang L, Yeow AYK (2021). Artificial intelligence in sepsis early prediction and diagnosis using unstructured data in healthcare. Nat. Commun..

[51] Liu XY, Wu JX, Zhou ZH (2009). Exploratory undersampling for class-imbalance learning. IEEE Trans. Syst. Man Cybern. B.

[52] Okuyama H, Kubota A, Oue T, Kuroda S, Ikegami R, Kamiyama M (2002). A comparison of the clinical presentation and outcome of focal intestinal perforation and necrotizing enterocolitis in very-low-birth-weight neonates. Pediatr. Surg. Int..

[53] Buchheit JQ, Stewart DL (1994). Clinical comparison of localized intestinal perforation and necrotizing enterocolitis in neonates. Pediatrics.

[54] Coates EW, Karlowicz MG, Croitoru DP, Buescher ES (2005). Distinctive distribution of pathogens associated with peritonitis in neonates with focal intestinal perforation compared with necrotizing enterocolitis. Pediatrics.

[55] Mintz AC, Applebaum H (1993). Focal gastrointestinal perforations not associated with necrotizing enterocolitis in very-low-birth-weight neonates. J. Pediatr. Surg..

[56] Murdoch WJ, Singh C, Kumbier K, Abbasi-Asl R, Yu B (2019). Definitions, methods, and applications in interpretable machine learning. Proc. Natl. Acad. Sci. USA..

[57] Rai A (2020). Explainable AI: From black box to glass box. J. Acad. Mark. Sci..

[58] Chang YS, Park H-Y, Park WS (2015). The Korean neonatal network: an overview. J. Korean Med. Sci..

[59] Chawla NV, Bowyer KW, Hall LO, Kegelmeyer WP (2002). SMOTE: Synthetic minority over-sampling technique. J. Artif. Intell. Res..

[60] Johnson JM, Khoshgoftaar TM (2019). Survey on deep learning with class imbalance. J. Big Data Ger..

[61] Kingma, D. P. & Ba, J. *Adam: A Method for Stochastic Optimization*. *CoRR.* http://arxiv.org/abs/1412.6980 (2015).

[62] Vasilev I, Slater D, Spacagna G, Roelants P, Zocca V (2019). Python Deep Learning: Exploring Deep Learning Techniques and neural Network Architectures with Pytorch, Keras, and TensorFlow.

[63] Pedregosa F, Varoquaux G, Gramfort A (2011). Scikit-learn: Machine learning in Python. J. Mach. Learn. Res..

