# Supplementary material for: Development of artificial neural networks for early prediction of intestinal perforation in preterm infants
Source: Sci Rep. 2022 Jul 15;12:12112. doi: 10.1038/s41598-022-16273-5 (PMC9287325; doi:10.1038/s41598-022-16273-5)
Supplement: Supplementary file 1 — Supplementary Tables. [file 41598_2022_16273_MOESM1_ESM.docx]

**Supplementary Table 1.** Performance metrics of proposed ANN models; When positive cases are oversampled in validation set

|  | AUROC | F1-score | PPV | NPV | Sensitivity | Specificity |
| --- | --- | --- | --- | --- | --- | --- |
| NEC | | | | | | |
| Model 1 | 0.8210 | 0.7701 | 0.7524 | 0.7779 | 0.7886 | 0.7404 |
| NEC-IP | | | | | | |
| Model 1 | 0.8665 | 0.7181 | 0.6721 | 0.8726 | 0.7708 | 0.8068 |
| Model 2 | 0.8834 | 0.8093 | 0.7862 | 0.8231 | 0.8338 | 0.7732 |
| Model 3 | 0.8692 | 0.7273 | 0.6452 | 0.8993 | 0.8333 | 0.7645 |
| SIP | | | | | | |
| Model 1 | 0.8498 | 0.8059 | 0.7166 | 0.8889 | 0.9205 | 0.6360 |
| Model 2 | 0.8799 | 0.8204 | 0.7237 | 0.9234 | 0.9470 | 0.6385 |
| Model 3 | 0.8633 | 0.8041 | 0.7926 | 0.8104 | 0.8160 | 0.7865 |

Abbreviations: ANN, artificial neural network; NEC, necrotizing enterocolitis; NEC-IP, intestinal perforation associated with necrotizing enterocolitis; SIP, spontaneous intestinal perforation; AUROC, area under the receiver operating characteristic curve; PPV, positive predictive value; NPV, negative predictive value

**Supplementary Table 2.** Performance metrics of proposed ANN models; When negative cases are undersampled in validation set

|  | AUROC | F1-score | PPV | NPV | Sensitivity | Specificity |
| --- | --- | --- | --- | --- | --- | --- |
| NEC | | | | | | |
| Model 1 | 0.8809 | 0.7819 | 0.8525 | 0.7590 | 0.7222 | 0.8750 |
| NEC-IP | | | | | | |
| Model 1 | 0.8785 | 0.7957 | 0.8222 | 0.7843 | 0.7917 | 0.7482 |
| Model 2 | 0.9028 | 0.8333 | 0.8333 | 0.8333 | 0.8333 | 0.8333 |
| Model 3 | 0.8863 | 0.7954 | 0.8750 | 0.7678 | 0.7292 | 0.8958 |
| SIP | | | | | | |
| Model 1 | 0.8760 | 0.8235 | 0.7447 | 0.8966 | 0.9211 | 0.6842 |
| Model 2 | 0.9176 | 0.8674 | 0.8000 | 0.9355 | 0.9474 | 0.7632 |
| Model 3 | 0.8829 | 0.8158 | 0.8158 | 0.8158 | 0.8158 | 0.8158 |

Abbreviations: ANN, artificial neural network; NEC, necrotizing enterocolitis; NEC-IP, intestinal perforation associated with necrotizing enterocolitis; SIP, spontaneous intestinal perforation; AUROC, area under the receiver operating characteristic curve; PPV, positive predictive value; NPV, negative predictive value

**Supplementary Table 3.** Variables and abbreviations used for machine learning

| Ordinal scaled variables  (4 variables) | Apgar score at 1 minute (apgs1); Apgar score at 5 minutes (apgs5); Degree of maternal education (medu); Degree of paternal education (fedu) |
| --- | --- |
| Continuous variables  (7 variables) | Gestational age (gagew, weeks); Birth weight (bwei, g); Maternal age (mage, year); Birth height (bhei, cm); Birth head circumference (bhead, cm); Body temperature at birth (btem, ℃); Hydrogen ion concentration in blood within 1 hour after birth (bbph) |
| Categorical variables  (43 variables) | Sex (sex); Multiple gestation (mulg); Pregnancy process (prep, natural pregnancy/in vitro fertilization); Gestational diabetes mellitus (Gdm); Overt diabetes mellitus (Odm); Pregnancy induced hypertension (pih); Chronic hypertension (Chtn); Histological chorioamnionitis (chor); Premature rupture of membrane (prom); Antenatal steroids (ster); Delivery mode (delm, virginal delivery/cesarean section); Oligohydramnios (Oamni); Polyhydramnios (Hamni); Need for initial resuscitation (resu); Need for oxygen supplementation at birth (resuo); Need for continuous positive airway pressure at birth (resup); Need for endotracheal intubation at birth (resui);); Need for cardiac massage at birth (resuh); Administering epinephrine at birth (resue); Gravida (gran); Parity (parn); Maternal country of origin (mcou); Paternal country of origin (fcou); Respiratory distress syndrome (rds); Need for surfactant (sft); Congenital bacterial or fungal infection (ibif); Air leak syndrome (als); Massive pulmonary hemorrhage (mph); Pulmonary hypertension (ph); Use of steroid for bronchopulmonary dysplasia (strdu); Indomethacin use for PDA (indopda); Ibuprofen use for PDA (ibupda); Low blood pressure within 7 days after birth (lbp); Inotropic use for low blood pressure (inolbp); Neonatal seizure (nese); Intraventricular hemorrhage grade III/IV (inhg); Sepsis (seps); Fungal infection (fsf1); Meningitis (meni); Use of prophylactic anti-fungal agent (antif); Transfusion (erythran) |
